# 1129. Outpatient Prescribing During the COVID-19 Pandemic

**DOI:** 10.1093/ofid/ofab466.1322

**Published:** 2021-12-04

**Authors:** Ganga Moorthy, Congwen Zhao, Michael J Smith

**Affiliations:** 1 Duke University, Durham, North Carolina; 2 Duke University Medical Center, Durham, North Carolina

## Abstract

**Background:**

The Joint Commission requires ambulatory healthcare systems to collect, analyze and report antimicrobial prescribing data. Duke University Health System (DUHS) piloted a dashboard to capture outpatient prescribing for pediatric patients with URI. Implementation in 2020 allowed for an assessment of antibiotic prescribing during the pandemic.

**Methods:**

We included patients 0 - < 19 years seen at DUHS for URI and pharyngitis from 1/1/2019 -2/21/2021. Patient characteristics included: age, sex, race, ethnicity, Pediatric Medical Complexity Algorithm (PMCA) score and insurance status (public versus private). Provider characteristics included: type (physician, NP, PA) and specialty (pediatrics, family medicine, internal medicine, other). We compared pre- and post-COVID ( March 1, 2020) prescribing and prescribing during telehealth versus in-person visits. A logistic regression model was used to identify factors independently associated with antibiotic prescribing.

**Results:**

62,447 children were seen during the study period, 29% of whom received an antibiotic. Amoxicillin was the most commonly prescribed antibiotic (64.4%), followed by cefdinir (11%) amoxicillin-clavulanic acid (10%) and azithromycin (8%). Factors associated with antibiotic prescribing are shown in Table 1. White race, private insurance, visits with nurse practitioners and visits with non-pediatric providers were associated with high prescribing. Higher PMCA scores, indicating greater medical complexity, were associated with decreased likelihood of prescribing. Although the total number of outpatient visits plummeted during the COVID period, rates of prescribing only decreased mildly from 31% to 25% (Figure 1).

Table 1. Factors Associated with Antibiotic Prescribing in Logistic Regression Model

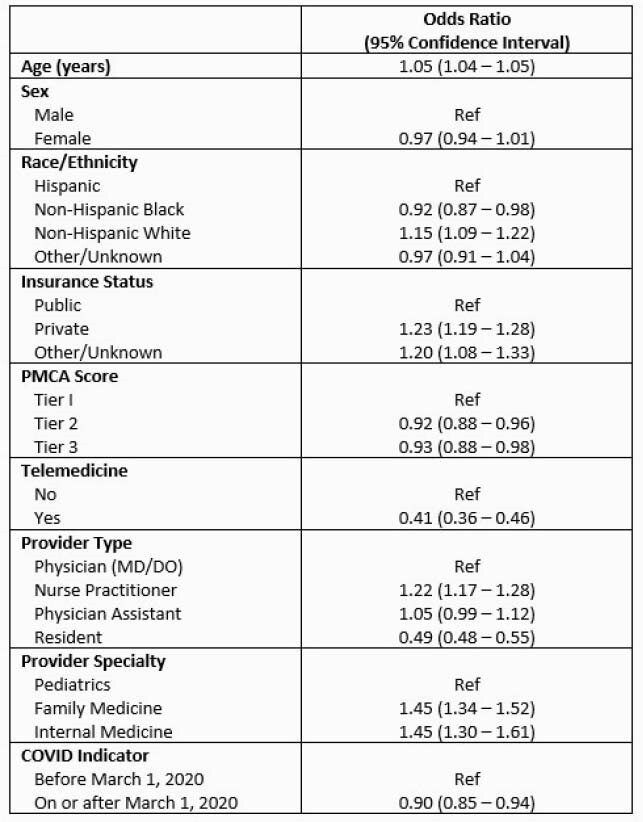

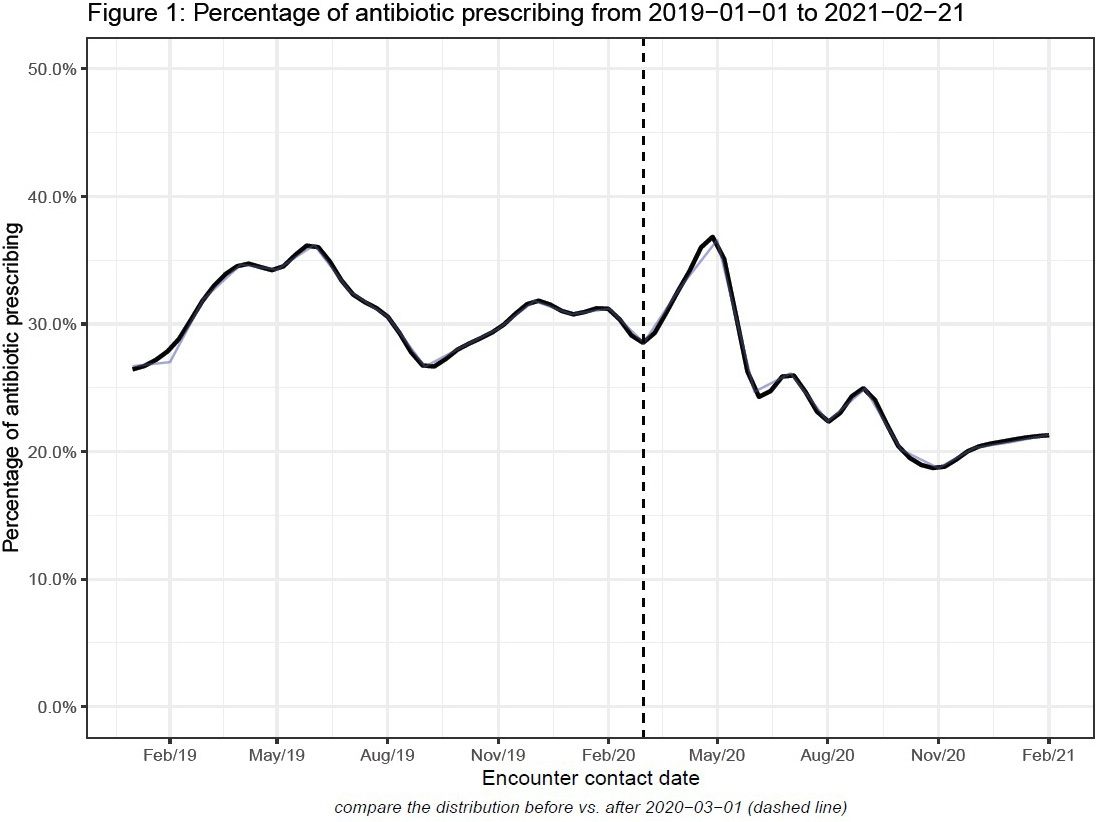

**Conclusion:**

Outpatient prescribing was associated with multiple patient and provider characteristics. Similar to other studies, white race, private insurance, and visits with non-physician, non-pediatric providers were associated with antibiotic prescription. Despite a large decrease in the number of outpatient visits during the pandemic, rates of prescribing for URI decreased minimally. A better understanding of factors associated with antibiotic prescribing during the pandemic may identify priority targets for outpatient stewardship as mitigation strategies are relaxed.

**Disclosures:**

**Michael J. Smith, MD, M.S.C.E**, **Merck** (Grant/Research Support)**Pfizer** (Grant/Research Support)

